# Pott's Spine with Bilateral Psoas Abscesses

**DOI:** 10.1155/2012/208946

**Published:** 2012-07-22

**Authors:** Sanjeevani Masavkar, Preeti Shanbag, Prithi Inamdar

**Affiliations:** Department of Pediatrics, Lokmanya Tilak Municipal Medical College & General Hospital, Sion, Mumbai 400022, India

## Abstract

A high degree of suspicion and appropriate imaging studies are required for the early diagnosis of Pott's spine. We describe a 4-year-old boy with Pott's disease of the lumbar spine with bilateral psoas abscesses. The child responded to conservative treatment with antituberculous treatment and ultrasonographically guided percutaneous drainage of the abscesses.

## 1. Introduction

Vertebral tuberculosis is the most common form of skeletal tuberculosis and is encountered most frequently in the first 3 decades of life, though it may occur at any age between 1 to 80 years [1]. A delay in diagnosis and initiation of treatment may cause severe and irreversible neurologic sequelae including paraplegia. We describe a 4-year-old boy with Pott's disease of the lumbar spine and bilateral psoas abscesses, who was treated successfully with antituberculous therapy and ultrasonographically guided percutaneous drainage of the psoas abscesses.

## 2. Case Report

A four-year-old Indian boy presented with a history of swelling and pain over the lumbar spine, low-grade fever, and failure to thrive for 6 months and abdominal distension for 3 months. The abdominal distension had increased since the last 5 days and was associated with a bulge in the left loin. There was a history of an abnormal gait with the child bending forward while walking. There was no weakness in the lower limbs nor were there any bladder symptoms. The mother had received antituberculous therapy for pulmonary tuberculosis 2 years ago. The child had been seen by various private practitioners in his village but was finally brought to Mumbai for investigation.

**Figure 1:**
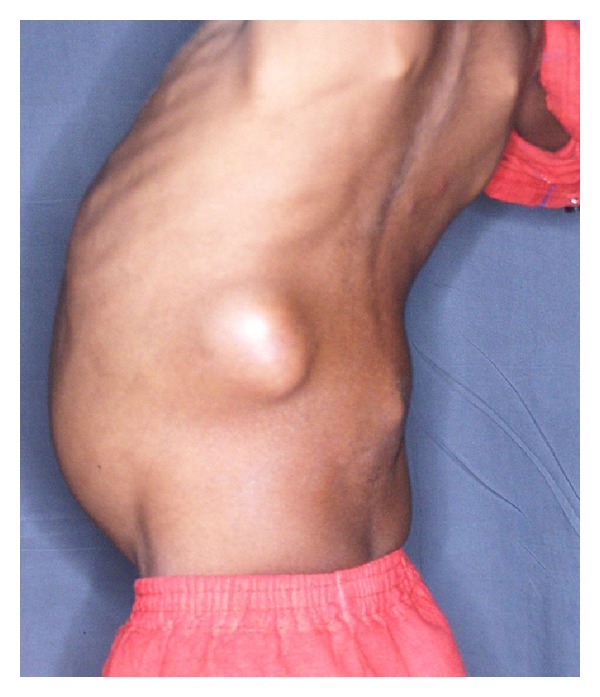
Photograph of child (posterolateral view) showing abdominal distension, swelling in left loin and gibbus.

Physical examination revealed an ill-looking pale child. His weight was 11.2 kg and height 89 cm. both below the 5th percentile for age. There were 3 left inguinal lymph nodes, matted and nontender, the largest of them being 2 cm by 3 cm in size. There was a gibbus at the level of L4-L5 vertebrae. The abdomen was distended with palpable bilateral large cystic masses. The left-sided mass was 20 cm by 10 cm, whereas the one on the right side was 15 cm by 10 cm in size. There was also a swelling in the left loin which was 6 cm by 8 cm in size (Figure 1). 

Central nervous system examination revealed a conscious child with normal higher functions and cranial nerves. Examination of the motor system revealed normal tone and power in both upper and lower limbs. The deep tendon reflexes were brisk in both lower limbs. The plantar reflexes were flexor bilaterally. Abdominal and cremasteric reflexes could be elicited normally. The sensory system was normal.

**Figure 2:**
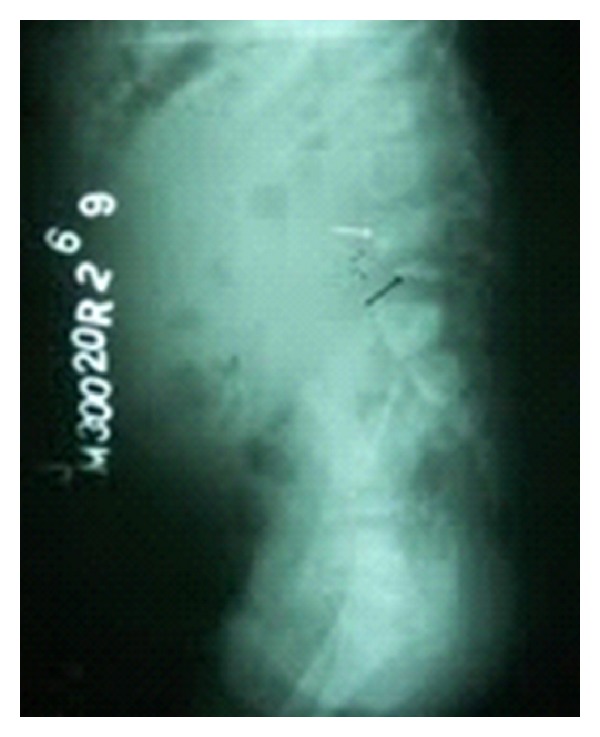
*X*-ray lumbosacral spine (lateral view) showing gibbus at L4-L5 level.

**Figure 3:**
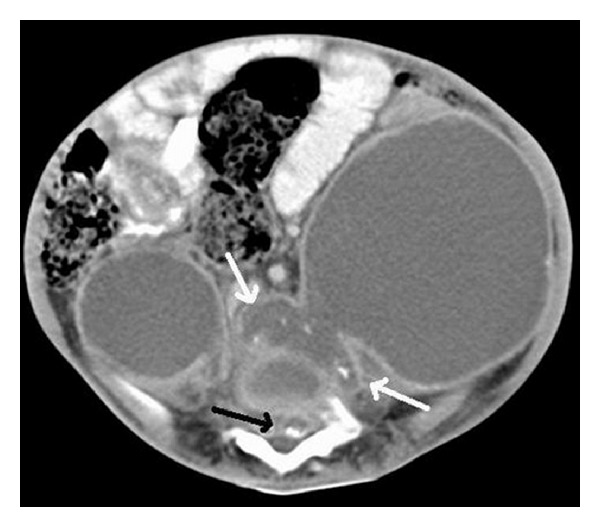
CT scan of abdomen and lumbar spine-axial section showing bilateral iliopsoas abscesses reaching up to the prevertebral region with a few hyperdense areas and bony fragments within (white arrows). Also seen is the epidural component in the spinal canal (black arrow).

Investigations showed hemoglobin of 7.1G/dL, a total WBC count of 15,000/cu·mm with a differential count of 25% lymphocytes and 75% polymorphs, and a platelet count of 6.4 × 10^5^/cu·mm. The erythrocyte sedimentation rate (ESR) was 80 mm at the end of 1 hour. The ELISA for HIV was negative. Lateral X-ray of the lumbosacral spine showed a wedge-shaped collapse of L4 vertebral body with bony fragments in the prevertebral space (Figure 2). Ultrasonography (USG) of the abdomen revealed bilateral large psoas abscesses with extension of the left psoas abscess through the left paraspinal muscle into the subcutaneous plane posteriorly. The computerized tomography (CT) scan of the abdomen (Figure 3) showed lesions of cystic density in both psoas muscles with peripheral enhancing walls and calcific areas within. There was destructive collapse of L4 vertebra with peri- and paravertebral abscesses communicating with the psoas abscess bilaterally. There was also an epidural abscess at L4-L5 level compressing the spinal cord. There was partial rotation of the right kidney with the pelvicalyceal system facing anteriorly. 

Ultrasonography-guided aspiration was undertaken on both sides using pigtail catheters and the drains were kept in situ. Thick white pus was drained (150 mL on the left side and 50 mL from the right side). Staining of the pus and culture for acid-fast bacilli was positive. Routine bacterial culture was sterile.

The patient was immobilized and started on antituberculous therapy with isoniazid (5 mg/kg/day), rifampicin (10 mg/kg/day), pyrazinamide (25 mg/kg/day), and ethambutol (20 mg/kg/day). Intravenous antibiotics ceftriaxone and amikacin were also started pending the report of bacterial culture. Three weeks later the USG showed almost complete resolution of the abscesses and the drains were removed. An external lumbar brace was provided to facilitate ambulation. With the above treatment, the patient showed a steady weight gain with decrease in the abdominal distention. At discharge on day 30, the child was afebrile, weighed 14 kg, and had no neurological deficit. At followup a month later, the child was gaining weight and had a normal gait.

## 3. Discussion

The opportunity for early diagnosis of spinal tuberculosis is often missed by health practitioners since symptoms are vague. The reported average duration of symptoms before diagnosis is 4 months but can be considerably longer [2]. This is due to the nonspecific presentation of chronic back pain which is the earliest and most common symptom. Constitutional symptoms such as weight loss, loss of appetite, and evening rise of temperature may occur [2]. 

Delay in diagnosis can be catastrophic in vertebral tuberculosis. Compression of the spinal cord can lead to severe neurological sequelae including paraplegia. Pott's disease most often affects the lower thoracic and lumbar spine while disease of the upper thoracic and cervical spine is more disabling. Neurological complications are more frequent when the upper and midthoracic spine is involved, as the spinal canal is narrowest between T3–T10 [2]. Cervical spine tuberculosis is characterized by pain and neck stiffness and patients may present with dysphagia or stridor [2]. 

Our patient despite having long-standing disease got away with no neurologic deficit since involvement of the spine was in the lumbar region. Cold abscesses may occur in long-standing cases and these may track their way through the intermuscular planes [2]. In our patient, the left psoas abscess had tracked posteriorly through the left paraspinal muscles into the subcutaneous plane in the left loin. 

Radiographic changes associated with Pott's disease present relatively late and include lytic destruction of anterior portion of vertebral body, increased anterior wedging, and enlarged psoas shadow with or without calcification. Intervertebral disks may be shrunk or destroyed and vertebral bodies may show variable degrees of destruction. Fusiform paravertebral shadows suggest abscess formation [3]. Computerised tomography (CT) scanning provides much better bony detail of irregular lytic lesions, sclerosis, disk collapse, and disruption of bone circumference. Low-contrast resolution provides a better assessment of soft tissue, particularly in epidural and paraspinal areas. Magnetic resonance imaging of the spine is the standard method of evaluation of disc space infection and is most useful in demonstrating extension into the soft tissues [4].

Conservative management with percutaneous drainage of the abscesses along with antitubercular treatment is recommended when vertebral lesions are located in one or two vertebrae with no serious disturbance in vertebral stabilization [5]. Indications for surgical treatment of Pott disease are neurologic deficit, spinal deformity with instability or pain, no response to medical therapy (continuing progression of kyphosis or instability), and large paraspinal abscess. In Pott's disease that involves the cervical spine, early surgical intervention is required due to the high frequency and severity of neurologic deficits. Also, severe abscess compression may induce dysphagia or upper airway obstruction [6].

A 4-drug regimen should be used to treat Pott's disease. Isoniazid and rifampin should be administered during the whole course of therapy.Additional drugs are administered during the first 2 months of therapy. These are generally chosen among the first-line drugs, which include pyrazinamide, ethambutol, and streptomycin. Therapy should be for a minimum duration of 6 months and could be extended up to 9 months depending upon the response [7]. 

In conclusion, Pott's spine should be considered in the differential diagnosis of chronic back pain in children so that treatment is initiated early and significant disability prevented.
